# Maternal Dietary Improvement or Leptin Supplementation During Suckling Mitigates the Long-Term Impact of Maternal Obesogenic Conditions on Inflammatory and Oxidative Stress Biomarkers in the Offspring of Diet-Induced Obese Rats

**DOI:** 10.3390/ijms252211876

**Published:** 2024-11-05

**Authors:** Catalina Amadora Pomar, Jenifer Trepiana, Irene Besné-Eseverri, Pedro Castillo, Andreu Palou, Mariona Palou, Maria P. Portillo, Catalina Picó

**Affiliations:** 1 Laboratory of Molecular Biology, Nutrition and Biotechnology (Group of Nutrigenomics, Biomarkers and Risk Evaluation), University of the Balearic Islands, 07122 Palma, Spain; c.pomar@uib.es (C.A.P.); pedro.castillo@uib.es (P.C.); andreu.palou@uib.es (A.P.); cati.pico@uib.es (C.P.); 2 Health Research Institute of the Balearic Islands (IdISBa), 07120 Palma, Spain; 3 CIBER Fisiopatología de la Obesidad y Nutrición (CIBEROBN), Instituto de Salud Carlos III (ISCIII), 28029 Madrid, Spain; jenifer.trepiana@ehu.eus (J.T.); irene.besne@ehu.eus (I.B.-E.); mariapuy.portillo@ehu.eus (M.P.P.); 4 Nutrition and Obesity Group, Department of Nutrition and Food Sciences, Faculty of Pharmacy, Lucio Lascaray Research Centre, University of the Basque Country (UPV/EHU), 01006 Vitoria, Spain; 5 BIOARABA Institute of Health, 01006 Vitoria-Gasteiz, Spain; 6 Artificial Intelligence Research Institute of the Balearic Islands (IAIB), 07122 Palma, Spain

**Keywords:** metabolic programming, inflammation, antioxidant defenses, maternal nutrition, leptin, lactation

## Abstract

This study investigates the impact of maternal nutrition during lactation on inflammation and oxidative stress in the offspring of diet-induced obese rats, along with the potential benefits of leptin supplementation during suckling. Dams were fed either a standard diet (SD), a western diet (WD) before and during gestation and lactation (WD-dams), or a WD switched to an SD during lactation (Rev-dams). Offspring were supplemented with leptin or vehicle during suckling and then fed an SD or WD until four months. Offspring of the Rev-dams exhibited improved metabolic indicators, including lower body weight, reduced plasma levels of TNF-alpha, a higher adiponectin/leptin (A/L) ratio, enhanced liver antioxidant defenses, and decreased inflammation markers in white adipose tissue (WAT) compared to WD-dams, with sex differences. Leptin supplementation further modulated these markers, reducing oxidative stress in liver and inflammation in WAT and liver (e.g., hepatic *Tnfa* expression decreased by 45% (males) and 41% (females) in the WD group on an SD), and improving the A/L ratio, with effects varying by maternal conditions and sex. In conclusion, this study underscores the importance of maternal nutrition and leptin intake during suckling in shaping long-term metabolic and inflammatory health in offspring, offering strategies to mitigate the adverse effects of maternal obesity on future generations.

## 1. Introduction

Inflammation and oxidative stress are critical factors influencing metabolic health. Chronic low-grade inflammation, often stemming from poor dietary habits, is closely associated with the development of metabolic disorders such as obesity, type 2 diabetes, and cardiovascular diseases [1]. Inflammatory mediators like cytokines can disrupt insulin signaling and promote insulin resistance, exacerbating metabolic dysfunction [1,2]. Additionally, oxidative stress, characterized by an imbalance between the production of reactive oxygen species (ROS) and antioxidant defenses, further contributes to cellular damage and inflammation [3]. In this context, nutrition plays a crucial role in modulating these processes and cellular responses, and both the liver and white adipose tissue (WAT) have been proposed as key tissues managing the inflammation and antioxidant response to nutritional conditions and metabolic status [4]. Balanced diets, rich in fruits, vegetables, and whole grains, can mitigate oxidative stress and inflammation, thereby supporting metabolic health and reducing the risk of related diseases [5]. Conversely, diets high in sugars and saturated fats can exacerbate these harmful processes, underscoring the critical intersection of nutrition, inflammation, and oxidative stress in metabolic health [6,7].

Likewise, the nutritional environment during prenatal and early postnatal life exerts a significant influence on later metabolic health [8,9], including programming the offspring’s inflammatory and oxidative stress responses [10,11]. Adequate nutrition during these pivotal periods can enhance the development of the fetal and neonatal immune systems, thereby reducing the risk of chronic inflammation and oxidative-stress-related pathologies in later life. Conversely, inadequate maternal nutrition can predispose offspring to a pro-inflammatory state and heightened oxidative stress [12], ultimately contributing to metabolic disorders [13]. In this context, there is substantial evidence showing that maternal obesity or consumption of an obesogenic diet increases the risk of obesity-related comorbidities in offspring [14,15]. However, improving the maternal diet during lactation in diet-induced obese rats mitigates many adverse programming effects in their offspring associated with maternal obesity [16]. This suggests that, in addition to the impact of maternal conditions during gestation, the maternal diet during lactation, rather than the obese state itself, plays a significant role in determining the future metabolic health of offspring [16].

In addition, certain bioactive compounds present in breast milk, such as leptin, are believed to play a crucial role during the suckling period, supporting the proper development of the neonate and having long-lasting effects in adulthood [17]. In rats, supplementation with physiological doses of leptin during the suckling period has been shown to improve metabolic health and prevent the development of obesity and related disorders later in life [18]. Leptin supplementation may also help reverse potential alterations caused by adverse perinatal conditions, such as poor nutrition during gestation due to maternal calorie restriction [17,19]. However, the programming effects of leptin ingested during the suckling period appear to depend on maternal dietary conditions, with benefits on metabolic parameters in adulthood being less evident in offspring of dams fed an obesogenic diet [20]. However, to our knowledge, the potential effects of leptin ingested during the suckling period on modulating inflammation status and antioxidant capacity in later life, and whether the effects depend on maternal conditions, have not been directly assessed.

It is hypothesized that improving the maternal diet during lactation in diet-induced obese dams, along with oral leptin supplementation in offspring during the suckling period, will reduce the negative effects of adverse perinatal conditions on the inflammatory and oxidative stress status of adult offspring. Thus, in this study, we aimed to assess the potential benefits of both improving maternal nutrition during lactation in diet-induced obese dams and the effects of leptin supplementation at physiological doses during the suckling period in mitigating the adverse effects of maternal perinatal obesogenic conditions on the inflammatory and oxidative stress status in offspring consuming either a standard diet (SD) or western diet (WD) from weaning until 4 months of age.

## 2. Results

### 2.1. Effects of Maternal Conditions and Leptin Treatment During Suckling on Phenotypic Traits in Adult Offspring

Table 1 shows the body weight, fat content, and weights of rWAT and liver of animals at four months of age. The body weight and fat content data were previously published [20]. The O-WD animals (males on an SD and both sexes, when considered together, on a WD) exhibited higher body weight compared to O-C and O-Rev animals, although without a significant increase in fat mass percentage. Nevertheless, O-WD males under SD conditions displayed greater rWAT depot weight than O-C animals. Of note, O-Rev rats on a WD displayed lower body weight than O-C animals. Furthermore, O-WD males and females under SD conditions displayed increased liver weight compared to O-C animals.

Regarding the long-term impact of leptin supplementation during suckling, the leptin-treated groups under SD conditions, when considered together, exhibited a lower fat mass percentage compared to their respective vehicle-treated counterparts, with the effect being particularly pronounced in O-Rev males (Mann–Whitney U test). Furthermore, leptin-treated females under SD conditions showed a decreased weight in the rWAT depot, especially in O-Rev animals (Mann–Whitney U test).

### 2.2. Effects of Maternal Conditions and Leptin Treatment During Suckling on Circulating Parameters in Adult Offspring

**Figure 1:**
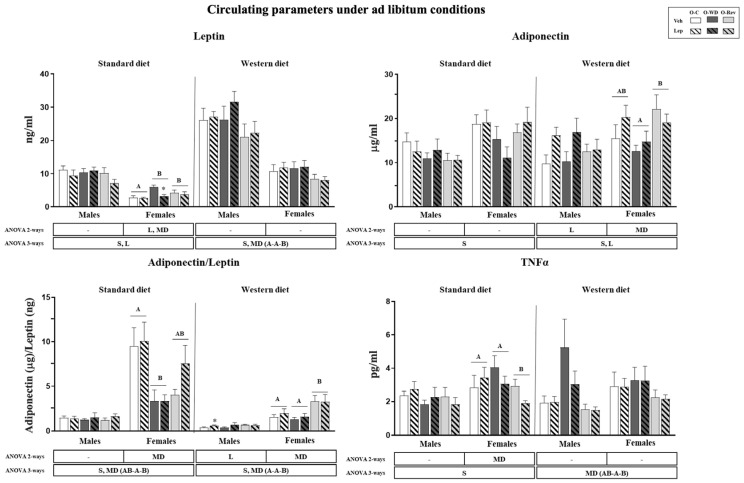
Circulating parameters of O-C, O-WD, and O-Rev male and female animals treated with vehicle or leptin during suckling and fed an SD or WD from weaning, at four months of age. Data are presented as the mean ± SEM (n = 8–12). Statistics: After data separation depending on post-weaning diet, three-way ANOVA was performed to analyze the effects of sex, maternal diet, and/or leptin treatment. In each sex, two-way ANOVA was performed to analyze the effects of leptin treatment and/or maternal diet. Single comparisons between leptin- and vehicle-treated rats of all experimental groups were carried out using the Mann–Whitney U test. Symbols: sex (S), maternal diet (MD), leptin treatment (L); Data that do not share a letter are significantly different, A ≠ B (*p* < 0.05, LSD post hoc, two- or three-way ANOVA); *, different from their vehicle-treated equal (*p* < 0.05, Mann–Whitney U test). Abbreviations: offspring of C-dams (O-C), offspring of WD-dams (O-WD), offspring of Rev-dams (O-Rev), vehicle (Veh), leptin (Lep).

Circulating levels of leptin, adiponectin, MCP-1, and TNF-alpha levels were assessed under ad libitum conditions in male and female O-C, O-WD, and O-REV animals, treated with vehicle or leptin during the suckling period, and subsequently fed a standard diet (SD) or western diet (WD) from weaning until 4 months. The A/L ratio was calculated as a potential marker of adipose tissue dysfunction and to estimate the cardiometabolic risk associated with obesity and metabolic syndrome [21]. The data are presented in Figure 1.

O-WD and O-Rev females on an SD displayed higher circulating levels of leptin than the O-C group. However, O-WD, but not O-Rev, females displayed a lower A/L ratio compared to O-C females, while O-Rev females exhibited lower TNF-alpha levels compared to O-C and O-WD rats. Under WD conditions, the O-Rev group (both males and females, when considered together) displayed lower circulating leptin levels and a higher A/L ratio than the O-C and O-WD groups. The increased A/L ratio was particularly marked and significant in females when analyzed by two-way ANOVA. O-Rev females also exhibited higher circulating adiponectin levels than the O-WD group. Furthermore, O-Rev animals under WD conditions (both males and females, when considered together) exhibited lower TNF-alpha levels compared to O-WD rats.

Regarding the effects of leptin treatment, under SD conditions, leptin-treated animals had lower circulating leptin levels, with the effect being particularly evident in females and especially in the O-WD group (Mann–Whitney U test). Under WD conditions, leptin-treated animals displayed higher adiponectin levels, with a more marked effect in males. Additionally, leptin-treated males showed a higher A/L ratio compared to their vehicle-treated counterparts, with the effect being more marked in O-C animals (Mann–Whitney U test).

### 2.3. Effects of Maternal Conditions and Leptin Treatment During Suckling on rWAT Gene Expression in Adult Offspring

**Figure 2:**
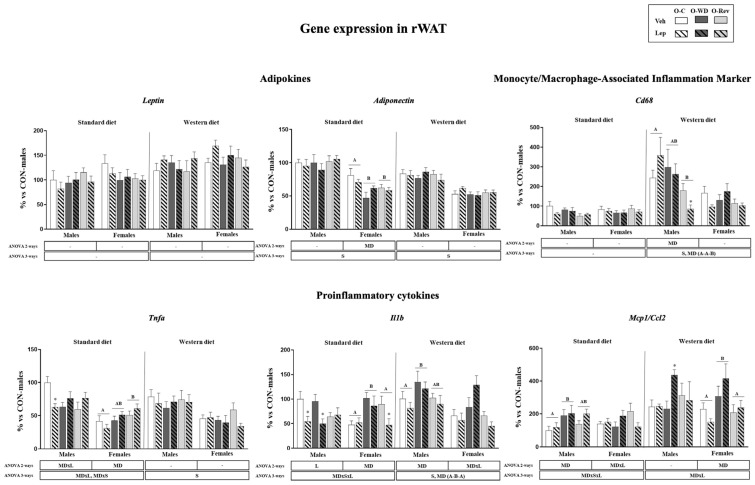
Expression levels of genes related to inflammation in retroperitoneal white adipose tissue of O-C, O-WD, and O-Rev male and female animals treated with vehicle or leptin during suckling and fed an SD or WD from weaning, at four months of age. Data are presented as the mean ± SEM (n = 8–12) and are expressed as a percentage of the value for the O-C male rats. Statistics: After data separation depending on post-weaning diet, three-way ANOVA was performed to analyze the effects of sex, maternal diet, and/or leptin treatment. In each sex, two-way ANOVA was performed to analyze the effects of leptin treatment and/or maternal diet. Single comparisons between leptin- and vehicle-treated rats of all experimental groups were carried out using the Mann–Whitney U test. Symbols: sex (S), maternal diet (MD), leptin treatment (L); Data that do not share a letter are significantly different, A ≠ B (*p* < 0.05, LSD post hoc, two- or three-way ANOVA); *, different from their vehicle-treated equal (*p* < 0.05, Mann–Whitney U test). Abbreviations: offspring of C-dams (O-C), offspring of WD-dams (O-WD), offspring of Rev-dams (O-Rev), vehicle (Veh), leptin (Lep).

Gene expression levels of selected genes related to inflammation in the rWAT are depicted in Figure 2. Regarding the effects of maternal conditions, under SD conditions, O-WD females displayed greater mRNA levels of *Il1b* than both the O-C and O-Rev groups, but O-Rev females displayed higher mRNA levels of *Tnfa* compared to the O-C group. Under WD conditions, O-WD rats showed higher expression levels of *Il1b* (both males and females, when considered together) and *Ccl2* (only females) than O-C rats, but levels were normalized to control levels in the O-Rev groups. Regarding adiponectin, O-WD and O-Rev displayed lower mRNA levels than O-C animals.

Regarding the effects of leptin treatment during suckling, under SD conditions, leptin-treated males displayed lower *Il1b* mRNA levels than the vehicle-treated groups, particularly the O-C and O-WD groups (Mann–Whitney U test). Leptin-treated O-Rev females also displayed lower mRNA levels of *Il1b* (Mann–Whitney U test). Additionally, leptin-treated O-C males displayed lower mRNA levels of *Tnfa* than their vehicle-treated counterparts (Mann–Whitney U test). Under WD conditions, leptin-treated males of the O-Rev group displayed lower mRNA levels of *Cd68*, whereas leptin-treated males of the O-WD group displayed greater mRNA levels of *Ccl2* than their vehicle-treated counterparts (Mann–Whitney U test).

### 2.4. Effects of Maternal Conditions and Leptin Treatment During Suckling on Hepatic Gene Expression in Adult Offspring

**Figure 3:**
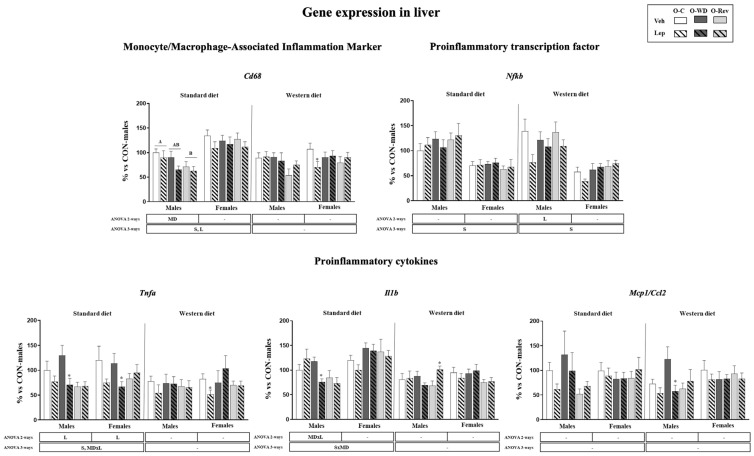
Expression levels of genes related to inflammation in liver of O-C, O-WD, and O-Rev male and female animals treated with vehicle or leptin during suckling and fed an SD or WD from weaning, at four months of age. Data are presented as the mean ± SEM (n = 8–12) and are expressed as a percentage of the value for the O-C male rats. Statistics: After data separation depending on post-weaning diet, three-way ANOVA was performed to analyze the effects of sex, maternal diet, and/or leptin treatment. In each sex, two-way ANOVA was performed to analyze the effects of leptin treatment and/or maternal diet. Single comparisons between leptin- and vehicle-treated rats of all experimental groups were carried out using the Mann–Whitney U test. Symbols: sex (S), maternal diet (MD), leptin treatment (L); Data that do not share a letter are significantly different, A ≠ B (*p* < 0.05, LSD post hoc, two- or three-way ANOVA); *, different from their vehicle-treated equal (*p* < 0.05, Mann–Whitney U test). Abbreviations: offspring of C-dams (O-C), offspring of WD-dams (O-WD), offspring of Rev-dams (O-Rev), vehicle (Veh), leptin (Lep).

Hepatic expression of selected genes related to inflammation is depicted in Figure 3. No marked effects were observed depending on the maternal diet. However, O-Rev males exposed to SD conditions displayed lower mRNA expression levels of *Cd68* compared to O-C animals. More pronounced effects were observed with leptin supplementation during suckling. In animals weaned onto an SD, leptin treatment resulted in decreased mRNA levels of *Cd68* and *Tnfa* in both males and females, with a more significant decrease in the case of *Tnfa* in the O-WD group (Mann–Whitney U test). Additionally, leptin-treated O-WD males under SD conditions displayed lower expression levels of the proinflammatory cytokine *Il1b* than vehicle-treated animals (Mann–Whitney U test).

Under WD conditions, *Cd68* mRNA levels were decreased in leptin-treated O-C females compared to their vehicle counterparts (Mann–Whitney U test). Notably, in males, leptin treatment resulted in decreased mRNA levels of *Nfkb* (two-way ANOVA) and *Ccl2* in the O-WD group (Mann–Whitney U test) compared to their vehicle-treated counterparts. However, leptin treatment resulted in increased expression levels of *Il1b* in males of the O-Rev group (Mann–Whitney U test).

### 2.5. Effects of Maternal Conditions and Leptin Treatment During Suckling on Hepatic Antioxidant Markers in Adult Offspring

**Figure 4:**
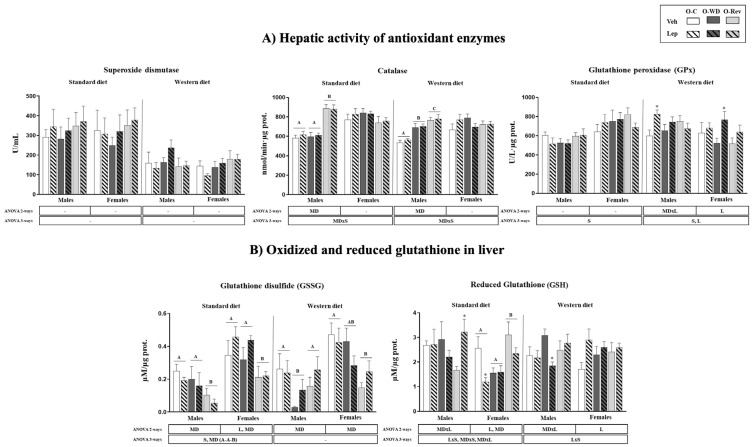
Markers of antioxidant defenses in the liver of O-C, O-WD, and O-Rev male and female animals treated with vehicle or leptin during suckling and fed an SD or WD from weaning, at four months of age. Data are presented as the mean ± SEM (n = 8–12). Statistics: After data separation, depending on the post-weaning diet, three-way ANOVA was performed to analyze the effects of sex, maternal diet, and/or leptin treatment. In each sex, two-way ANOVA was performed to analyze the effects of leptin treatment and/or maternal diet. Single comparisons between leptin- and vehicle-treated rats for all experimental groups were carried out using the Mann–Whitney U test. Sex (S), maternal diet (MD), and leptin treatment (L); Data that do not share a letter are significantly different, A ≠ B ≠ C (*p* < 0.05, LSD post hoc and two- or three-way ANOVA); * different from their vehicle-treated equal (*p* < 0.05, Mann–Whitney U test). Offspring of C-dams (O-C), offspring of WD-dams (O-WD), offspring of Rev-dams (O-Rev), vehicle (Veh), and leptin (Lep).

Hepatic antioxidant capacity was assessed using enzymatic and nonenzymatic markers, as shown in Figure 4. No significant differences in superoxide dismutase (SOD) activity were observed among the experimental groups. However, hepatic catalase activity was increased in O-Rev males compared to O-C and O-WD males, both under SD and WD conditions. Additionally, O-WD males exhibited increased catalase activity compared to O-C males under WD conditions. Interestingly, leptin treatment during suckling led to an increase in hepatic glutathione peroxidase (GPx) activity in animals under WD conditions, with a more pronounced effect observed in O-C males and O-WD females (Mann–Whitney U test).

The levels of the oxidized and reduced forms of glutathione (glutathione disulfide (GSSG) and GSH, respectively) are depicted in Figure 4B. Under SD conditions, the O-Rev group showed lower GSSG levels (both males and females) and higher GSH levels (females) compared to the O-C and O-WD rats. Under WD conditions, hepatic GSSG levels were also reduced in O-Rev females compared to O-C females. Conversely, among males, the O-WD group displayed lower GSSG levels compared to both the O-C and O-Rev animals.

Leptin treatment during suckling was associated with increased GSSG levels and decreased GSH levels in females on an SD compared to their respective vehicle-treated group. Conversely, on a WD, leptin-treated females exhibited higher GSH levels compared to their vehicle-treated counterparts. Among males, only O-Rev leptin-treated animals displayed increased GSH levels under SD conditions (Mann–Whitney U test), whereas, on a WD, only O-WD leptin-treated animals exhibited decreased GSH levels in comparison to their respective vehicle-treated group (Mann–Whitney U test).

### 2.6. Overall Effects of Maternal Conditions and Leptin Treatment During Suckling

**Figure 5:**
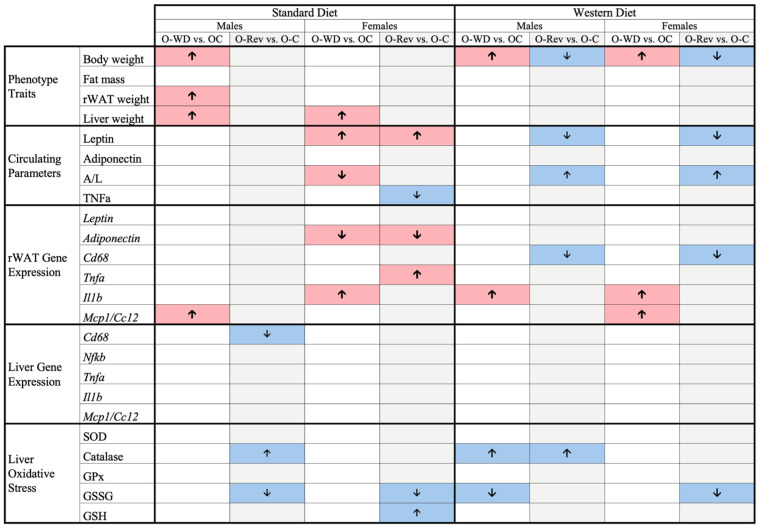
Summary of the main long-term effects of maternal conditions during suckling on O-C, O-WD, and O-Rev male and female animals weaned onto an SD or WD. Offspring of C-dams (O-C), offspring of WD-dams (O-WD), and offspring of Rev-dams (O-Rev). Arrows indicate increases (↑) or decreases (↓) according to a two-way or three-way ANOVA. Red arrows represent negative effects, while blue arrows indicate positive effects.

**Figure 6:**
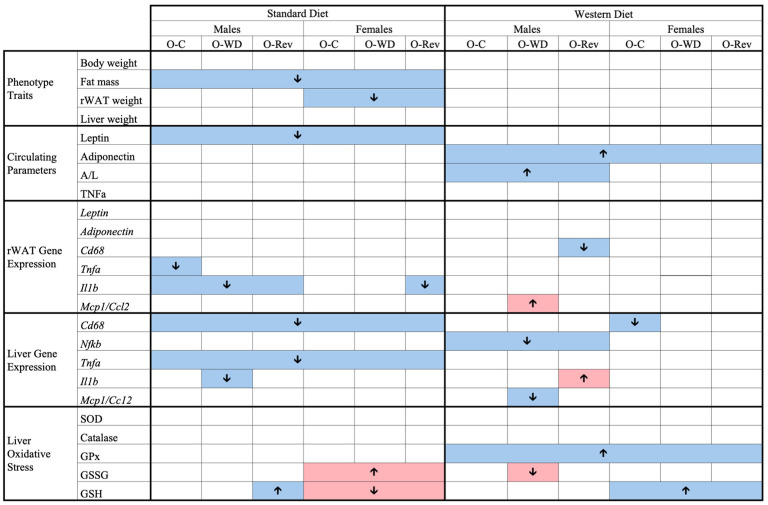
Summary of the main long-term effects of leptin supplementation during suckling on O-C, O-WD, and O-Rev male and female animals weaned onto an SD or WD. Offspring of C-dams (O-C), offspring of WD-dams (O-WD), and offspring of Rev-dams (O-Rev). Arrows indicate increases (↑) or decreases (↓) according to a Mann–Whitney U test, two-way or three-way ANOVA. Red arrows represent negative effects, while blue arrows indicate positive effects.

To provide an overview of the results, the main long-term effects of maternal conditions and leptin supplementation during suckling are summarized in Figure 5 and Figure 6, respectively.

## 3. Discussion

The present study examines the long-term impact of maternal nutritional conditions during perinatal life, along with the effects of a daily leptin supplement administered to offspring at physiological doses during the suckling period, on inflammatory and oxidative stress biomarkers in adult rats.

Dietary patterns and obesity are widely recognized to influence inflammatory and oxidative stress processes in human and animal studies [6,22,23,24]. Moreover, maternal obesity and/or obesogenic diet consumption during the perinatal period has also been shown to program offspring for higher susceptibility to alterations in oxidative and inflammatory health, affecting insulin sensitivity and overall metabolic health [12,25,26]. Studies in this regard have focused mainly on the effects of maternal diets high in fats, which have been shown to cause maternal inflammation that is then transmitted to offspring through the activation of various inflammatory pathways [10]. However, corrective strategies to reverse the metabolic malprogramming have been less studied. They have generally been aimed at being applied during pregnancy and have shown some controversial results [10,27,28,29]. We previously demonstrated that maternal obesity, resulting from a diet rich in fats and simple sugars (the so-called WD) during the perinatal period, led to adverse effects on the metabolic parameters of the offspring [20]. These effects were particularly evident when animals were subsequently exposed to a WD. However, a balanced maternal diet during lactation, despite the dams maintaining excess adiposity, was able to reverse or attenuate most of these metabolic alterations [20]. Here, in the same cohort of animals, we show that maternal-diet-induced obesity also influences markers of inflammation and oxidative stress in adult offspring, with effects shown even after a long feeding period with a healthy diet. Importantly, implementing a healthy diet in dams during lactation mitigated most of the adverse effects on these markers in adult offspring, even when they were weaned onto a WD.

The liver and WAT are primary tissues involved in obesity-related inflammation and oxidative stress. Their dysfunction not only contributes to local tissue damage but also has systemic effects that exacerbate metabolic disorders [2,4]. Moreover, a crosstalk link between both tissues has been recognized, where they communicate through the release of adipokines and cytokines and influence metabolic status [2,4]. WAT, in addition to being the primary energy reservoir, secretes a variety of hormones and adipokines that regulate energy balance, insulin sensitivity, and inflammatory processes [30]. In the context of obesity, WAT acts as a significant source of pro-inflammatory cytokines, leading to the chronic low-grade inflammation characteristic of this metabolic status [30,31]. Additionally, the infiltration of immune cells, particularly macrophages, into WAT exacerbates the inflammatory state [30,31], creating a vicious cycle of inflammation and metabolic dysregulation. Thus, the balance of pro- and anti-inflammatory signals from WAT is crucial in maintaining metabolic health and insulin sensitivity.

Leptin and adiponectin are two hormones primarily produced by WAT, playing key roles in the regulation of energy balance and insulin sensitivity [32]. The circulating levels of these hormones are positively (for leptin) and negatively (for adiponectin) correlated with body fat mass and act as pro- and anti-inflammatory hormones, respectively [4,33,34]. The adiponectin/leptin (A/L) ratio may serve as an indicator of inflammation status and has been proposed as a promising index to estimate adipose tissue dysfunction and as a marker of cardiometabolic risk [21,34]. Notably, maternal WD feeding before gestation and throughout gestation and lactation resulted in a decreased A/L ratio in female offspring maintained on the SD diet. These animals (females) also exhibited other markers of inflammation in the adipose tissue, including elevated *Il1b* mRNA expression and decreased adiponectin mRNA expression, while males showed increased *Mcp1* mRNA expression. This may indicate that the metabolic malprogramming caused by adverse maternal conditions during pregnancy and lactation cannot be reversed, or not completely, by following a balanced diet after weaning and thereafter. Other studies in animals have shown that a high-fat diet during pregnancy induces inflammation in tissues, as well as features of the metabolic syndrome, in adult offspring, independent of environmental conditions and postnatal diet [11], in line with our present results. Interestingly, normalizing the maternal diet during lactation mitigated the effects of adverse maternal conditions during gestation on most of these markers. Moreover, consistent with the improved profile of circulating inflammatory markers, these animals showed lower body weight (in males) and a reduction in tissue weights of the liver (in both sexes) and rWAT (in males) when fed the SD, compared to the offspring of dams exposed to a WD for the entire period (gestation and lactation). Furthermore, when exposed to a WD after weaning, O-Rev animals exhibited decreased leptin levels and an increased A/L ratio compared to controls, along with lower body weight. Moreover, these animals were also protected from the presence of increased mRNA expression levels of pro-inflammatory cytokines in rWAT, including *Il1b* and the monocyte chemoattractant factor *Mcp1* observed in the offspring of WD-fed dams, and also exhibited, compared to controls, lower expression levels of *Cd68,* the known macrophage infiltration marker [35]. This suggests that improving maternal nutrition during lactation may even protect offspring from the adverse effects of consuming an obesogenic diet after weaning, which may also be related to the previously reported enhanced sensitivity to leptin and insulin [20]. Similarly, normalization of the maternal diet during lactation ameliorated the circulating profile of the recognized mediator of inflammation TNF-alpha [36]. Specifically, TNF-alpha levels decreased in O-Rev females under an SD and in both sexes of this group under a WD, compared to the O-WD group, supporting the idea of a certain protection against diet-induced obesity. However, the increased mRNA levels of *Tnfa* in WAT of O-Rev females on an SD, though seemingly contradictory to the proposed protective effects of a healthy maternal diet during lactation, could be tentatively interpreted as a compensatory response to the decreased circulating levels of TNF-alpha in these animals. TNF-alpha plays a crucial role in regulating various cellular processes, including immune function, inflammation, and cell proliferation and differentiation, thus maintaining appropriate levels is essential for ensuring cellular homeostasis [36].

The pro-inflammatory state in offspring caused by maternal WD feeding during gestation and lactation could be explained, in part, through changes in milk composition. In this regard, we have previously described significant differences in the lipid profile, including an increased ratio of pro-inflammatory arachidonic acid to anti-inflammatory eicosapentaenoic acid and docosahexaenoic acid, in the milk of WD-fed dams [37]. Improving the maternal diet during lactation prevented most of these alterations and was accompanied by a normalization of the offspring’s plasma lipid profile [37]. Moreover, dams of the Rev group also displayed lower expression levels of pro-inflammatory biomarkers and higher mRNA expression of adiponectin in the mammary gland, compared to dams fed a WD during gestation and lactation [37]. Therefore, improving the maternal diet during lactation may reverse this pro-inflammatory imprinting by improving the maternal inflammatory state and normalizing milk composition.

The liver is a crucial organ for detoxification and metabolism. Obesity and unbalanced diets rich in fat favor excess lipid accumulation in the liver and represent a major risk for the development and progression of metabolic dysfunction-associated steatotic liver disease (MASLD) [38]. This lipid deposition triggers an inflammatory response characterized by increased production of pro-inflammatory cytokines like TNF-alpha, IL-6, and IL-1β [38]. Additionally, the liver’s metabolic processes can generate ROS, contributing to oxidative stress [38]. Interestingly, we show here that a balanced maternal diet during lactation also exerted positive effects in the liver of the offspring, either fed an SD or WD after weaning, by promoting their antioxidant defenses, in particular on the activity of catalase and improving the content of oxidized and reduced glutathione. Catalase is a crucial antioxidant enzyme in the liver that catalyzes the decomposition of hydrogen peroxide (H_2_O_2_) into water and oxygen. This function is vital for protecting liver cells from oxidative damage caused by the accumulation of H_2_O_2_ [39]. Glutathione exists in the following two main forms: the nonenzymatic antioxidant reduced glutathione (GSH), and oxidized glutathione or glutathione disulfide (GSSG). These forms play a central role in the antioxidant defense system and cellular redox homeostasis, acting in concert with other redox-active compounds [40]. Therefore, the increased antioxidant capacity in the liver shown by the offspring of Rev-dams is suggested as one of the mechanisms by which these animals exhibited improved metabolic health. The impact of the maternal diet on the expression of inflammatory markers in the liver was less apparent than in rWAT. Nonetheless, it is worth noting that O-Rev male animals displayed lower mRNA expression levels of the macrophage infiltration marker *Cd68* in the liver compared to O-WD males under the SD, consistent with the positive effects of a healthy maternal diet during lactation.

Taken together, our results, both at the level of circulating parameters and gene expression in the liver and rWAT, demonstrate that adverse maternal conditions during critical periods of development have profound effects on the programmed predisposition to inflammation and oxidative stress in the offspring. However, while a change to a healthy diet beginning in juvenile age (after weaning) and continuing until adulthood is not sufficient to reprogram this susceptibility, our results reveal that a healthy maternal diet during lactation, even in the presence of maternal obesity or excessive body fat accumulation in dams, can reverse or significantly mitigate the detrimental programming effects of adverse maternal conditions during pregnancy on offspring health.

Leptin, a hormone naturally ingested within breast milk, has been recognized as an essential modulator of later body weight and metabolic homeostasis [17,20]. The action of leptin during the suckling period has been primarily attributed to its neurotrophic effects, as evidenced by animal studies [41,42]. This action may partly explain the lasting beneficial effects on metabolic programming when leptin is supplemented during suckling to offspring of well-nourished dams, gestational energy-restricted dams, or diet-induced obese dams [18,19,20]. However, the effects of leptin intake during suckling on inflammation and antioxidant responses have not been directly explored. Notably, we previously reported in the same cohort of animals that leptin supplementation during suckling exerted a protective effect on metabolic health, particularly in males fed an SD after weaning [20]. However, maintaining a maternal WD throughout the perinatal period, combined with an obesogenic diet after weaning, diminished the positive effects of leptin treatment on insulin sensitivity, especially in females [20].

The present study suggests significant effects of leptin supplementation in WD-fed offspring on the A/L ratio and the hepatic antioxidant capacity, even greater than those observed on an SD, with sex differences. Specifically, on an SD, animals supplemented with leptin exhibited a reduction in body fat along with reduced plasma leptin levels and decreased inflammatory markers in the liver. This aligns with the previously observed improved metabolic profile, which included increased insulin sensitivity, regardless of maternal diet, though this effect was only seen in males [20]. The sex difference is also evident here. O-Rev males treated with leptin showed a greater hepatic reserve of GSH than vehicle-treated rats, indicating an enhanced capacity to respond to oxidative stress under SD conditions. In contrast, leptin-treated females on an SD showed a reduced GSH reserve. Notably, on a WD, leptin-treated males exhibited increased plasma adiponectin levels and higher A/L ratio, lower hepatic expression of the pro-inflammatory transcription factor *Nfkb*, and increased activity of the antioxidant enzyme GPx compared to the vehicle-treated counterparts. This suggests that, although WD led to increased fat accumulation in the liver [20], animals supplemented with physiological doses of leptin during suckling had an enhanced capacity to reduce both the inflammatory response and oxidative stress caused by the fat surplus. This aligns with the previously proposed idea that leptin-treated animals exhibit a better adaptive response to hypercaloric diets [20,43], likely due to their greater tendency to channel excess dietary energy toward WAT, which could be related to increased insulin sensitivity. In this context, the higher circulating levels of the adipokine adiponectin along with the increased A/L ratio in leptin-treated males compared to the vehicle group are noteworthy. Adiponectin is known to enhance the liver’s anti-inflammatory and antioxidant capacity [44], partly by downregulating *Nfkb* expression [45]. Moreover, diet-induced obesity typically results in decreased adiponectin levels [46] and increased oxidative stress [47,48]. For instance, mice fed a high-fat diet have been shown to exhibit a significant reduction in antioxidant defenses in the liver, including a decrease in the activity of GPx [22], a critical enzyme that helps protect cells from oxidative damage by reducing peroxides using GSH as a substrate [49]. Furthermore, adiponectin deficiency has been associated with increased liver tumor formation and oxidative stress in mice with nonalcoholic steatohepatitis [50]. Additionally, inhibition of both the secretion and expression of adiponectin has been observed after treating adipocytes in cell culture with ROS [51]. Collectively, these observations suggest that the increase in adiponectin levels due to leptin supplementation during the suckling period may be one mechanism by which the liver’s ability to manage excessive energy intake is improved in males. This improvement likely occurs through enhanced antioxidant defenses and a reduced inflammatory response. On the other hand, while leptin supplementation in females has previously been associated with worsened insulin sensitivity and increased hepatic lipid accumulation (in the O-WD group) compared to vehicle-treated animals [20], the current findings reveal an increased antioxidant capacity in the liver in these animals. This might suggest an adaptive response to the stress caused by the obesogenic diet, which could potentially confer long-term benefits. All in all, these findings provide further insights into the mechanisms by which leptin ingested during the suckling stage may program a lower predisposition to develop metabolic alterations in response to various challenges, with sex differences.

Taken together, present findings support potential strategies during lactation to reduce the risk of obesity and metabolic disorders in humans, potentially breaking the cycle of obesity transmission that significantly contributes to its rising prevalence worldwide [52]. However, a limitation of this study is the inherent differences between the rat model and human physiology, particularly regarding the timing and magnitude of metabolic responses [53,54]. While fundamental mechanisms such as energy balance regulation, hormonal signaling, and hypothalamic development are conserved across mammals, the exact outcomes may differ between species. Therefore, further research is imperative to validate these results in humans, with future studies focusing on enhancing the translatability of these findings. Additionally, whether the observed benefits of improving maternal diet or leptin supplementation during lactation translate into an improved phenotype at older ages, including behavioral and cognitive outcomes, merits further investigation.

In summary, this study highlights the complex interactions between maternal diet, leptin treatment during the suckling period, and long-term health outcomes in adult offspring. Both the normalization of maternal diet during lactation in diet-induced obese dams and leptin treatment during early development in pups appear to be potential interventions for positively influencing most of the metabolic and inflammatory parameters analyzed in the adult offspring, with varying effects based on sex, maternal diet, and offspring’s diet later on. In particular, maternal WD feeding before gestation and during gestation and lactation resulted in detrimental effects on the inflammatory response and antioxidant defenses in their adult offspring. The improvement in maternal diet during lactation was able to reverse most of these alterations in both male and female offspring. Furthermore, rat pups that received an oral physiological dose of leptin during suckling exhibited increased antioxidant capacity in the liver and decreased inflammatory markers in plasma, liver, and rWAT compared to those treated with the vehicle. While some differences were observed based on maternal conditions and sex, the effects were generally more pronounced under WD feeding conditions, associated with increased adiponectin levels and, in males, a higher A/L ratio, suggesting an enhanced capacity to counteract the harmful effects of an obesogenic diet. This study underscores the significance of maternal nutrition and early leptin intervention in shaping long-term metabolic and inflammatory health outcomes in offspring and highlights potential strategies to mitigate the adverse effects of maternal obesity on coming generations. Future studies exploring the mechanistic pathways underlying these sex-specific and diet-related differences, as well as their potential long-term effects on the aging process will be highly relevant.

## 4. Materials and Methods

### 4.1. Animals and Experimental Design

The animal protocol was subject to review and approval by the Bioethics Committee of the University of the Balearic Islands (Exp. 2018/13/AEXP, 23 January 2019) and was in accordance with the university’s policies on the use and care of laboratory animals.

The experimental design has been previously described [18,20]. In brief, virgin female Wistar rats, housed at 22 °C, with a light–dark period of 12/12 h and free access to food and water, were distributed into the following two groups: rats fed a standard chow diet (SD; 3.3 kcal·g^−1^, with 8.4% calories from fat, 72.4% from carbohydrates, and 19.3% from protein; Safe, Augy, France); and rats fed a high-fat and high-sucrose diet (WD, western diet, Research Diets, New Brunswick, USA; 4.7 kcal·g^−1^, with 40.0% calories from fat, 43.0% from carbohydrates, and 17.0% from proteins) from one month before coupled with male rats. The diets’ compositions are summarized in Supplementary Table S1.

Pregnant dams were housed individually and maintained on their pre-assigned diets throughout gestation. On the day after partum (PND1), litters were equated to 10 pups per dam (5 pups from each sex, if possible). During the lactation period, SD-fed dams continued with this diet (C-dams, n = 8). Of those fed the WD, half continued with the WD (WD-dams, n = 9), while the other half were switched to the SD (Rev-dams, n = 10). From PND1 to PND20, the offspring of C-, WD-, and Rev-dams (referred to as O-C, O-WD, and O-Rev) were orally supplemented with leptin (PeproTech, London, UK) at a dose equivalent to five times the dose ingested normally from breast milk [17] or the vehicle (water). Weaning was set at PND21, after which the animals were divided into the following two groups: one fed an SD and the other fed a WD (n = 8–12 per group) until sacrifice at the age of 4 months. Animals were decapitated under ad libitum feeding state. Blood samples (in heparinized tubes), liver, and retroperitoneal white adipose tissue (rWAT) were collected. Body weight and food intake were monitored.

### 4.2. Determination of Blood Parameters Under Ad Libitum Fed Conditions

Circulating parameters in plasma were determined at four months of age, under ad libitum feeding conditions. Plasma was obtained from blood samples by centrifugation (1000× *g*, 10 min, 4 °C). Enzyme-linked immunosorbent assay (ELISA) kits were used to determine adiponectin, leptin, and tumor necrosis factor alpha levels (TNF-alpha) (R&D Systems, Minneapolis, MN, USA).

### 4.3. Parameters Related to Oxidative Stress in Liver

Superoxide dismutase (SOD) was assessed spectrophotometrically using a commercial kit (Superoxide Dismutase Activity Assay Kit CS009, Sigma-Aldrich, St. Louis, MO, USA). The assay is based on creating superoxide anions by the xanthine–xanthine oxidase system. This measurement involved quantifying the reduction in WST-1 formazan formation. SOD functions by quenching superoxide anions, leading to the conversion of WST-1 into WST-1 formazan, being its absorbance measured. Catalase activity was determined spectrophotometrically following the procedure described by Aebi [55], wherein the disappearance of H_2_O_2_ at 240 nm was monitored. The activity of glutathione peroxidase (GPx) was also assessed by measuring its H_2_O_2_ scavenging capacity using a colorimetric commercial kit (Sigma-Aldrich, San Louis, MO, USA). The GPx levels in the samples were determined using a standard curve obtained with NADPH. The concentration of reduced glutathione (GSH) and oxidized glutathione (GSSG) in rat liver homogenates was colorimetrically assessed using a commercial kit (Sigma-Aldrich, San Louis, MO, USA). GSH measurement relies on the glutathione recycling system in the presence of GSH and DTNB fluorophore. In the case of GSSG, it can be measured by scavenging all existing GSH by 1-methyl-2-vinylpyridinium triflate as a scavenger reagent.

In all instances, an Infinite 200Pro plate reader (Tecan, Männedorf, Zurich, Switzerland) was used.

### 4.4. RNA Extraction

Total RNA was extracted from liver and rWAT using the commercial E.Z.N.A. Total RNA Kit I (Omega Bio-Tek, Inc., Norcross, GA, USA), quantified using the spectrophotometer NanoDrop ND-1000 (NanoDrop Technologies, Inc., Wilmington, DE, USA), and its integrity confirmed by 1% agarose gel electrophoresis.

### 4.5. Real-Time Quantitative Polymerase Chain Reaction (RT-qPCR) Analysis

RT-qPCR was used to measure mRNA expression levels of adiponectin, leptin, C-C motif chemokine ligand 2 (*Ccl2*), interleukin 1 beta (*Il1b*), tumor necrosis factor alpha (*Tnfa*), *Cd68* molecule (*Cd68*), and nuclear factor kappa B (*Nfkb*) in liver and rpWAT, as previously described [16]. Guanosine diphosphate dissociation inhibitor (*Gdi*) was selected as the reference gene. The relative expression of each mRNA was calculated as in [56].

### 4.6. Statistical Analysis

Data are presented as the mean ± SEM. First, data were separated according to the offspring diet (OD), and differences between experimental groups were analyzed by factorial analysis of variance (ANOVA), considering three factors (three-way ANOVA): sex (S), maternal diet (MD), and leptin treatment (L). Next, after the separation of the data by the OD and S of animals, differences due to two factors (MD and/or L) were analyzed by two-way ANOVA. Finally, when an interactive effect between MD and L was found, data were also separated by L, and a one-way ANOVA was performed to detect differences due to the MD in each treatment. All ANOVA tests were followed by a least significant difference (LSD) post hoc test when the effect of MD was significant. Single comparisons between the leptin-treated groups and vehicle-treated groups were carried out by the Mann–Whitney U test. The Shapiro–Wilk and Bartlett tests were used to evaluate the normality and homogeneity of variances of the data, respectively. Analyses were carried out with SPSS for Windows (SPSS, Chicago, IL, USA), with the threshold of significance set at *p* < 0.05.

## Figures and Tables

**Table 1:** Phenotypic traits at the age of four months.

		Males							Females							
		O-C		O-WD		O-Rev		ANOVA	O-C		O-WD		O-Rev		ANOVA	ANOVA
		Veh	Lep	Veh	Lep	Veh	Lep	2-way	Veh	Lep	Veh	Lep	Veh	Lep	2-way	3-way
**Body** **weight** **(g)**	**SD**	434 ± 11	423 ± 11	455 ± 10	470 ± 18	423 ± 10	400 ± 12	**MD A-B-A**	246 ± 6	249 ± 4	259 ± 4	258 ± 6	240 ± 7	249 ± 9	**-**	**SxMD**
	**WD**	513 ± 14	535 ± 19	546 ± 19	570 ± 23	528 ± 26	507 ± 19	**-**	283 ± 7	291 ± 7	280 ± 11	292 ± 10	276 ± 11	271 ± 6	**-**	**S, MD A-B-C**
**Fat** **mass** **(%)**	**SD**	13.6 ± 1.2	11.3 ± 1.1	12.0 ± 1.0	13.2 ± 1.0	12.2 ± 0.9	10.1 ± 0.5 *	**-**	13.8 ± 1.1	13.6 ± 0.6	13.6 ± 0.7	11.9 ± 0.8	13.1 ± 0.9	12.0 ± 0.9	**-**	**L**
	**WD**	22.4 ± 1.3	24.3 ± 1.9	27.8 ± 2.3	27.9 ± 2.2	24.9 ± 2.3	23.9 ± 1.0	**-**	22.6 ± 0.9	24.2 ± 1.6	21.7 ± 2.2	23.8 ± 2.4	20.5 ± 2.0	19.4 ± 1.4	**-**	**S**
**rWAT** **weight** **(g)**	**SD**	8.6 ± 0.8	7.3 ± 0.7	9.2 ± 0.8	9.9 ± 0.9	8.3 ± 0.9	6.7 ± 0.6	**MD A-B-A**	3.4 ± 0.3	3.0 ± 0.1	3.1 ± 0.3	2.9 ± 0.2	3.0 ± 0.3	2.4 ± 0.2 *	**L**	**SxMD**
	**WD**	18.8 ± 1.4	22.7 ± 1.3	22.4 ± 1.7	25.9 ± 3.1	24.1 ± 2.6	21.1 ± 2.0	**-**	5.7 ± 0.3	6.7 ± 0.5	6.5 ± 0.8	6.4 ± 0.7	5.6 ± 0.4	5.2 ± 0.4	**-**	**S**
**Liver** **weight** **(g)**	**SD**	12.3 ± 0.5	12.5 ± 0.4	13.2 ± 0.5	13.8 ± 0.8	12.4 ± 0.4	11.6 ± 0.6	**MD A-B-A**	6.6 ± 0.3	6.5 ± 0.3	7.6 ± 0.3	7.4 ± 0.3	6.9 ± 0.3	7.3 ± 0.4	**MD A-B-AB**	**S, MD A-B-A**
	**WD**	17.3 ± 0.6	17.9 ± 0.9	17.9 ± 0.9	18.3 ± 1.1	17.7 ± 0.9	16.5 ± 1.1	**-**	8.3 ± 0.1	8.4 ± 0.5	7.5 ± 0.5	7.8 ± 0.3	8.4 ± 0.3	7.9 ± 0.2	**-**	**S**

Body weight, fat content, and weights of rWAT and liver of O-C, O-WD, and O-Rev male and female animals treated with vehicle or leptin during suckling and fed an SD or WD from weaning, at four months of age. Data are presented as the mean ± SEM (n = 8–12). Statistics: After data separation, depending on post-weaning diet, a three-way ANOVA was performed to analyze the effects of sex, maternal diet, and/or leptin treatment. For each sex, a two-way ANOVA was performed to analyze the effects of leptin treatment and/or maternal diet. Single comparisons between leptin- and vehicle-treated rats for all experimental groups were carried out using the Mann–Whitney U test. Symbols: sex (S), maternal diet (MD), and leptin treatment (L); Data that do not share a letter are significantly different, A ≠ B ≠ C (*p* < 0.05, LSD post hoc and two- or three-way ANOVA); * different from their vehicle-treated equal (*p* < 0.05, Mann–Whitney U test). Offspring of C-dams (O-C); offspring of WD-dams (O-WD); offspring of Rev-dams (O-Rev); vehicle (Veh); leptin (Lep).

## Data Availability

Data are contained within the article or Supplementary Material.

## Supplementary Materials

The following supporting information can be downloaded at: https://www.mdpi.com/article/10.3390/ijms252211876/s1.
