# Supplementary material for: Maternal Dietary Improvement or Leptin Supplementation During Suckling Mitigates the Long-Term Impact of Maternal Obesogenic Conditions on Inflammatory and Oxidative Stress Biomarkers in the Offspring of Diet-Induced Obese Rats
Source: Int J Mol Sci. 2024 Nov 5;25(22):11876. doi: 10.3390/ijms252211876 (PMC11594198; doi:10.3390/ijms252211876)
Supplement: Supplementary file 1 [file ijms-25-11876-s001.zip › ijms-3267665-supplementary.pdf]

Supplementary Table S1. Composition of standard diet (SD) and western diet (WD).

**Description**	**Standard diet (SD)**	**Western diet (WD)**
**Energy density (kcal/g)**	3.33	4.68
**Macronutrient composition (%; g/100g)**		
Carbohydrates	60.4 (3.2 of sugars)	50.0 (35.0 of sugars)
Lipids	3.1	21.0
Proteins	16.1	19.8
Others (fiber, micronutrients and water)	21.4	9.4
**Caloric information (%; Kcal/100 Kcal)**		
Carbohydrates	72.4	40.0
Lipids	8.4	43.0
Proteins	19.3	17.0
